# Factors associated with social isolation and loneliness in community-dwelling older adults during pandemic times: a cross-sectional study

**DOI:** 10.1590/0037-8682-0195-2020

**Published:** 2021-07-02

**Authors:** Maycon Sousa Pegorari, Caroline de Fátima Ribeiro Silva, Fabrícia Coelho de Araújo, Juliana de Souza da Silva, Daniela Gonçalves Ohara, Alessandro Pena Matos, Areolino Pena Matos, Ana Carolina Pereira Nunes Pinto

**Affiliations:** 1 Universidade Federal do Amapá, Curso de Fisioterapia, Macapá, AP, Brasil.; 2 Hospital Ofir Loyola, Belém, PA, Brasil.

**Keywords:** Covid-19, Physical distancing, Older adults, Social isolation, Loneliness

## Abstract

**INTRODUCTION:**

Psychosocial aspects need to be discussed in the context of the Covid-19 pandemic. Currently, no studies have investigated the factors associated with social isolation and loneliness among community-dwelling older adults. Therefore, this study analyzed the association of social isolation and loneliness with socioeconomic, clinical, and health characteristics, and Covid-19-related variables, among community-dwelling older adults during the pandemic.

**METHODS:**

A cross-sectional study was conducted via a telephone survey of community-dwelling older adults aged ≥60 years in Macapa, Amapa, Brazil. A structured form was used to collect data. Descriptive and inferential analyses were performed using Pearson's correlation test and a linear regression model.

**RESULTS:**

Participants comprised 86 community-dwelling older adults with a mean age of 71.78+6.98 years. Among them, 9.3% were diagnosed with Covid-19, of whom 3.5% were hospitalized. Most participants reported no difficulty obtaining food, medicines, or attending routine medical appointments during the pandemic. Furthermore, 23.3% (n=20) were socially isolated, and 20.9% (n=18) reported feelings of loneliness. The mean values for fear, anxiety, and obsession were 19.01±7.25, 1.01±1.90, and 2.84±3.28, respectively. A moderate positive correlation was identified between loneliness and the number of diseases, and a weak positive correlation between loneliness and the number of medications and depressive symptoms and risk for sarcopenia. The linear regression model indicated that higher loneliness scores were associated with a greater number of diseases (β=0.288; p=0.007).

**CONCLUSIONS:**

The findings suggest a probable resilience of the older population to Covid-19, despite the association of loneliness with many diseases in times of a pandemic.

## INTRODUCTION

The coronavirus disease 2019 (Covid-19) pandemic, caused by the severe acute respiratory syndrome coronavirus 2 (SARS-CoV-2), has triggered social and economic crises and overwhelmed health services worldwide, including in Brazil^1^. Infection causes acute respiratory distress syndrome, damage to the myocardium, renal and liver complications, and can lead to death in its most severe forms. Older adults and those with comorbidities are generally more vulnerable to complications and present a higher risk of mortality when infected with SARS-CoV2^2^.

Currently, few pharmacological interventions have been shown to be effective in the treatment of Covid-19^3^. Although some recently developed vaccines provide significant protection against Covid-19^4^, maintaining physical distancing is still necessary until effective vaccination coverage is achieved^2^. Long-term social distancing measures may disproportionately affect older individuals^2^, resulting in greater isolation of institutionalized older people and several adverse health outcomes, posing a serious public health concern. In addition, many healthy community-dwelling older adults may not have strategic knowledge on how to adapt and manage their daily routines to the changes imposed by the pandemic. This situation exposes older adults to an increased risk of worsening pre-existing comorbidities^2^ and may have a negative impact on their psychosocial well-being and functionality.

Loneliness is a particularly concerning issue arising from the social isolation of older adults. It is associated with depression, cognitive decline, disability, cardiovascular diseases, increased institutionalization, and mortality in older adults^5^. Although these negative consequences are more prevalent among institutionalized and hospitalized older people, in the face of the pandemic, they may also be a concern for community-dwelling older adults.

Recent studies have found positive correlations between loneliness and worsening mental health and fitness^6^, as well as loneliness and social isolation and poor sleep quality^7^. Other studies have described the relationship between loneliness and social isolation with a decline in cognitive function^8^, and the role of loneliness as a mediator of the impact of social isolation on cognitive functioning in community-dwelling older adults^9^. It has been argued that home isolation could substantially reduce levels of moderate and vigorous physical activity, contributing to increased sedentary behavior. Physical inactivity induced by the Covid-19 pandemic, in its turn, could harm the cardiovascular health of individuals^10^.

While extraordinary efforts have been made to find effective treatments and vaccines for fighting Covid-19, the psychosocial aspects have yet to be thoroughly considered. Few studies, to our knowledge, have investigated the association between social isolation and loneliness in community-dwelling older adults. Investigating this relationship could provide evidence on which to base future health policy strategies for older populations and clinical decision-making processes. Therefore, the purpose of this study was to analyze the association of social isolation and loneliness with socioeconomic, clinical, and health characteristics, as well as Covid-19-related variables (obsession, anxiety, and fear) in community-dwelling older adults during the pandemic. 

## METHODS

### Type of study and sample

With a cross-sectional and analytical design, a telephone survey was conducted in June 2020 with community-dwelling older adults from from Macapa, Amapa, Brazil. The Ethics Committee of the Federal University of Amapa approved this study under opinion no. 4.100.122, before commencement, and the informed consent form was sent to the participants via WhatsApp or e-mail. Two copies of the informed consent were sent to the homes of the participants who did not have these resources to get their signatures. The sample size was estimated using the Power Analysis and Sample Size (PASS) application, version 13. With an a priori determination coefficient R^2^ = 0.13, in a linear regression model with four predictors, significance level or type I error of α = 0.05, and statistical power of 80% or type II error of β = 0.2, a minimum of 85 subjects were found to be needed for a representative sample of this population. The main outcome variable was the loneliness score on the Loneliness Scale of three items from the University of California, Los Angeles (UCLA).

Adults aged 60 years or over who lived in the urban area of Macapa were included. Subjects who were not found after three contact attempts via telephone and who were institutionalized and/or hospitalized were excluded. Using a database from a previous population-based survey carried out in 2017 in that municipality, a total of 411 community-dwelling older adults were contacted via telephone. Of these, seven had died, 26 had refused to undergo the interview, and 292 had not been found after three contact attempts (missed calls or missing numbers). Thus, 86 older adults were included in the study.

### Data collection instruments

#### Socioeconomic characteristics

Socioeconomic characteristics included the following variables: sex (male and female), age (in years), education (in years), marital status (with or without a partner), living arrangement (alone or accompanied), education (in years), individual monthly income (no income, up to one salary, two to three salaries, and four or more salaries).

#### Clinical and health characteristics

For clinical and health characteristics, the analyzed variables were as follows:

*Hospitalization in the last year (yes/no), occurrence of falls in the last year (yes/no), and fear of falling (yes/no);*

*Exposure time to sedentary behavior: Assessed with the questions: “How much time, in total, do you spend sitting on a weekday?” and "How much time do you spend sitting on a weekend day?"* ^11^. The average time spent sitting was estimated in min/day.

*Frailty:* Evaluated using the FRAIL scale, which consists of five yes or no questions assessing the presence of fatigue, muscular endurance, aerobic capacity, disease load, and weight loss. One point was given to each affirmative answer. The score ranges from 0 to 5 points, and the individuals are classified as robust (0 points), pre-frail (1 to 2 points), or frail (3 points)^12^.

*Risk of sarcopenia:* Assessed using SARC-F, this instrument contains five items that analyze individuals’ perception of their strength, ability to walk, rise from a chair, climb stairs, and experiences with falls. The diagnosis of probable sarcopenia was confirmed by positive SARC-F screening (score ≥4)^13^.

*Identification of vulnerable older adults:* Assessed using the Vulnerable Elders Survey 13 (VES - 13), which Maia et al^14^ cross-culturally adapted to Portuguese. The questionnaire consists of four parts: (1) age, (2) one question on self-rated health, (3) six questions on physical activities, and (4) five questions on activities of daily living. The maximum score is 10 points and values ​​greater than or equal to 3 indicate vulnerability.

#### Psychosocial characteristics

*Loneliness*: Assessed using the UCLA three-item Loneliness Scale, which measures the frequency and intensity of feelings of loneliness. The total score ranges from 3 to 9, with a higher score indicating higher levels of loneliness^15^. Older adults with a score ≥ 6 were classified as lonely^16^.

*Social isolation:* The Lubben Social Network Scale (LSNS-6) was used, which comprises a set of three questions assessing family ties and a comparable set of three questions assessing friendship ties. The total score ranges from 0 to 30. Subjects with scores below 12 were classified as socially isolated^17^.

*Depressive symptoms:* Measured using the Abbreviated Geriatric Depression Scale (GDS-4), this version is used to screen for depression in primary care services and consists of four closed questions with objective answers (yes or no)^18^. The scores ranged from 0 to 4 points. 

### Variables related to Covid-19

Four questions were extracted from the questionnaire for assessing the impact of the Covid-19 pandemic and accompanying mitigation efforts on older adults (QAICPOA) to assess issues related to obtaining food, medicines, and medical care in times of the pandemic^19^. Subjects were also asked about the possible diagnosis of Covid-19 and the need for hospitalization during the infection period.

*Obsession:* Assessed using the Obsession with Covid-19 Scale (OCS). A total OCS score ≥ 7 indicates that the subjects’ thinking about Covid-19^20^ is likely dysfunctional.

*Fear*: Assessed using the 7-item Fear of Covid-19 Scale (FCV-19S). The minimum possible score for each item was 1, and the maximum score was 5. The total score ranged from 7 to 35 points^21^.

*Anxiety:* Measured using the Coronavirus Anxiety Scale (CAS). A total CAS score ≥ 9 indicates probable coronavirus-related dysfunctional anxiety^22^.

### Data analysis

Descriptive and inferential statistics were used for the analyses. Correlation analysis between variables was performed using Pearson's correlation coefficient. A linear regression model was used to consider the statistical and theoretical criteria for the inclusion of potential predictors associated with the outcomes of interest. A 95% confidence interval (CI) and 5% significance level was used. The minimum prerequisites of normality, linearity, and homoscedasticity of residuals, as well as the absence of multicollinearity, were considered. All data were analyzed using the Statistical Package for Social Sciences version 21.0.

## RESULTS

A total of 86 older adults were interviewed. The mean age was 71.78 ± 6.98 years, and the majority were female (68.6%). Socioeconomic, clinical, and health characteristics are shown in Tables 1 and 2, respectively. In this study, 9.3% (n= 8) of the older adults reported a positive diagnosis of Covid-19, of whom 3.5% (n = 3) were hospitalized. The majority of respondents reported no difficulty obtaining food (73.3%), medicines (69.8%), or attending routine medical appointments (53.5%) during the pandemic period. Among the study participants, 20.9% (n = 18) reported feelings of loneliness, and 23.3% (n = 20) were socially isolated. The means for the Covid-19-related variables such as fear, anxiety, and obsession were 19.01 ± 7.25, 1.01 ± 1.90, and 2.84 ± 3.28, respectively (Table 3).

**TABLE 1:** Socioeconomic characteristics of community-dwelling older adults (n=86). Macapa, Amapa, Brazil (2020).

	Mean	SD	Median	Observed Variation
Age (years)	71.78	6.98	69.50	[62-100]
Sex (n/%)				
Male	27 (31.4)			
Female	59 (68.6)			
Education (years)	6.45	4.88	5.00	[0-20]
Individual income* (n/%)				
No income	5 (5.8)			
Up to 1 minimum wage	52 (60.5)			
2 to 3 minimum wages	26 (30.2)			
4 or more minimum wages	3 (3.5)			
Living arrangement (n/%)				
Accompanied	77 (89.5)			
Alone	9 (10.5)			
Marital Status (n/%)				
Without partner	52 (60.5)			
With partner	34 (39.5)			

*Current minimum wage in Brazil (2020): R$ 1,045.00; **SD:** Standard Deviation.

**TABLE 2:** Clinical and health characteristics of community-dwelling older adults (n=86). Macapa, Amapa, Brazil (2020).

Variables	Mean	SD	Median	Observed variation
Number of diseases	2.65	2.02	2.00	[0-11]
Number of medications	2.08	2.00	2.00	[0-11]
Hospitalizations in the last year (n/%)				
Yes	11 (12.8)			
No	75 (87.2)			
Falls in the last year (n/%)				
Yes	22 (25.6)			
No	64 (74.4)			
Fear of falling (n/%)				
Yes	56 (65.1)			
No	30 (34.9)			
Sedentary behavior (hours)	5.16	1.97	4.57	[1.57-10.57]
VES-13	2.60	3.00	1.00	[0-10]
Frailty (FRAIL Scale) (n/%)				
Not frail	36 (41.9)			
Pre-frail	40 (46.5)			
Frail	10 (11.6)			
Sarcopenia (SARC-F - score ≥4) (n/%)				
Yes	9 (10.5)			
No	77 (89.5)			
Depressive symptoms (GDS-4)	0.87	0.82	1.00	[0-4]

*SD: Standard Deviation; **VES:** Vulnerable Elders Survey; **GDS:** Geriatric Depression Scale.

**TABLE 3:** Psychosocial and Covid-19-related variables in community-dwelling older adults (n=86). Macapa, Amapa, Brazil (2020).

Variables	Mean	SD	Median	Observed Variation
Covid-19 diagnosis (n/%)				
Yes	8 (9.3)			
No	78 (90.7)			
Hospitalization for Covid-19 (n/%)				
Yes	3 (3.5)			
No	83 (96.5)			
Length of hospital stay (days)	0.20	1.15	0.00	[0-8]
Difficulty in obtaining food (n/%)				
None	63 (73.3)			
Some	10 (11.6)			
Much	8 (9.3)			
Unable or very difficult	5 (2.8)			
Difficulty in obtaining medicine (n/%)				
None	60 (69.8)			
Some	13 (15.1)			
Much	7 (8.1)			
Unable or very difficult	6 (7.0)			
Difficulty with getting routine medical care (n/%)				
None	46 (53.5)			
Some	12 (14.0)			
Much	17 (19.8)			
Unable or very difficult	11 (12.8)			
Fear (*Fear of Covid-19 Scale)*	19.01	7.25	18.00	[7-35]
Anxiety (CAS)	1.01	1.90	0.00	[0-9]
Obsession (OCS)	2.84	3.28	2.00	[0-14]
Loneliness (Loneliness Scale)	4.50	1.41	4.00	[3-9]
Loneliness (Loneliness Scale) (n/%)				
Yes	18 (20.9)			
No	68 (79.1)			
Social Isolation (LSNS-6)	17.84	6.12	16.00	[4-30]
Social Isolation (LSNS-6) (n/%)				
Yes	20 (23.3)			
No	66 (76.7)			

**SD:** Standard Deviation; **CAS:** Coronavirus Anxiety Scale; **OCS:** Obsession with Covid-19 Scale; **LSNS-6:** The Lubben Social Network Scale-6.

A moderate positive correlation between loneliness and the number of diseases and a weak correlation between loneliness and the number of medications, depressive symptoms (GDS-4), and risk for sarcopenia (SARC-F) were identified (Table 4). No correlations were found between the other variables (data not shown). In the linear regression analysis, the highest loneliness scores were associated with the highest number of diseases (β = 0.288; p = 0.007) (Table 5).

**TABLE 4:** Correlation of loneliness with clinical and health variables in community-dwelling older adults (n=86). Macapa, Amapa, Brazil (2020).

Variables	Number of diseases		Number of medications		Depressive symptoms (GDS-4)		Sarcopenia (SARC-F)	
	r	p	r	p	r	p	r	p
Loneliness (TILS Score)	0.334	0.002	0.235	0.029	0.248	0.021	0.251	0.020

**TILS:** Three-item Loneliness Scale; **GDS:** Geriatric Depression Scale; r = Pearson correlation coefficient; p<0.05.

**TABLE 5:** Linear regression analysis of loneliness and depressive symptoms, number of diseases, sarcopenia, and living arrangements in community-dwelling older adults (n=86). Macapa. Amapa, Brazil (2020).

Variables	Loneliness (TILS score)						
	B	SE	β	t	p	95% CI	
						Lower limit	Upper limit
GDS-4 Score	0.319	0.182	0.186	1.754	0.083	-0.043	0.681
Number of diseases	0.201	0.073	0.288	2.758	0.007	0.056	0.346
Sarcopenia (SARC-F)	0.118	0.103	0.126	1.145	0.256	-0.087	0.323
Living arrangements (alone)	-0.150	0.468	-0.033	-0.321	0.749	-1.082	0.782

**TILS:** Three-item Loneliness Scale; **GDS:** Geriatric Depression Scale; **SE:** Standard Error; **β:** beta coefficient; **CI:** Confidence Interval; p<0.05.

## DISCUSSION

This is one of the few studies to analyze the relationship between psychosocial, clinical, and health variables and loneliness and social isolation in a community-dwelling older population during the Covid-19 pandemic. In this study, 20.9% of the participants reported feelings of loneliness, and 23.3% were socially isolated. Additionally, this study found that higher loneliness scores were associated with a greater number of diseases.

Since the beginning of the pandemic, there have been significant concerns regarding the psychosocial consequences of physical distancing in older adults^2^. In the present study, the majority of participants did not report feelings of loneliness and reported low Covid-19-related fear, anxiety, and obsession. Nonetheless, attention should be paid to population groups, such as the older adults, who may be more vulnerable to the effects of physical distancing. Forced social isolation affected daily activity routines and interrupted socialization cycles, activities that were previously carried out in groups such as meeting in religious temples, access to informal and formal health care services, and opportunities for physical activity monitoring in health clinics, increasing the vulnerability and exposure of older people to health risks^5^. Some researchers claim that social isolation can significantly increase physical and mental health problems in older adults^23^. It should be highlighted that even a small increase in loneliness can pose a greater risk for poor health outcomes in older people, as it may raise the risk of anxiety and depression, physical health problems, and death^5^.

Our findings are in line with those of a previous study, which found that individuals with chronic conditions were at a higher risk of loneliness^24^. However, our results contradict previous studies that found that those who live alone would also be at a higher risk of loneliness^25^. It is worth noting that these studies were conducted in non-pandemic times, which may account for the differences between their results and ours. In the context of the coronavirus pandemic, as individuals with comorbidities are at a greater risk of complications from Covid-19, it is reasonable for them to take increased precautions, including isolating themselves from other people. Nevertheless, a social connection can persist despite physical distancing, especially when the use of digital technologies such as video calls may help reduce loneliness or social isolation^26^. For those who had previously lived alone, adaptations to physical distancing may have already occurred, and feelings of loneliness may not persist.

It is noteworthy that we did not have available data before the pandemic. As our interviews were conducted in June, physical distancing changes may have already occurred before the commencement of the study. A previous study^27^ assessed loneliness and social connection in undergraduates and a community sample before and during the Covid-19 pandemic. The authors noted no substantial change in social connections over time^27^, but they did notice a decrease in loneliness. Similar results were found in another study that examined the trajectory of loneliness in response to Covid-19 and found no significant increase in loneliness but a remarkable resilience of the participants to Covid-19^28^. It should be noted that these samples were not composed of older populations, and as previous studies have found that older adults reported lower levels of loneliness when compared to younger adults, future longitudinal studies are necessary to clarify whether loneliness follows a similar trend in older adults^29^.

Another hypothesis is the perceived social support and coping strategies of older adults. A previous cross-sectional survey conducted from March to April 2020 found that higher perceived social support was associated with lower loneliness^25^. Although we did not formally evaluate coping strategies, most participants in this study reported having a strong connection with spiritual aspects, including feelings of faith and hope, in addition to relying on family bonds. These aspects may have influenced their perspectives and feelings while facing the Covid-19 pandemic and they may have raised mechanisms to overcome this challenging situation. Indeed, social support from faith communities has generally been found to be positively associated with mental health^30^. A recent study conducted during the Covid-19 pandemic found that positive religious coping, intrinsic religiosity, and trust in God are strongly correlated with less stress and a more positive impact of Covid-19 on life^31^. Further studies are needed to explore coping strategies and their relationship with social isolation and loneliness in older adults during the Covid-19 pandemic.

Surprisingly, the present study found a weak correlation between loneliness and a few variables, including depressive symptoms, the number of medications taken, and the risk of sarcopenia. These results are in line with previous studies that found loneliness to be associated with depressive symptoms, anxiety, and dementia in older adults^5^ ^,^ ^32^. Additionally, loneliness is associated with declines in motor function, malnutrition, systemic arterial hypertension, and frailty^6^, all of which increase the risk of sarcopenia and use of medications. 

These results should be interpreted with caution, as this study included self-reports from older adults on their perception of emotional/sentimental issues, and the hypothesis of a trend for minimizing possible unfavorable results regarding emotional health should be considered. This study was conducted with community-dwelling older adults, and no formal diagnosis of mood disorders was obtained. Finally, as we live in an unprecedented public health crisis and little research has been conducted in this field, sparse comparative data are available. As the current situation evolves, further longitudinal studies monitoring loneliness over time are necessary to analyze the long-lasting impact of physical distancing in older adults.

The results of this study suggest a probable resilience of the older population to Covid-19, despite the association of loneliness with the number of diseases during the pandemic.
